# Characterization of the Ferroptosis-Related Genes for Prognosis and Immune Infiltration in Low-Grade Glioma

**DOI:** 10.3389/fgene.2022.880864

**Published:** 2022-04-26

**Authors:** Xiuwei Yan, Hang Ji, Zhihui Liu, Shuai Ma, Jiawei Dong, Xiaoyan Jiang, Xueyan Hu, Fang Wang, Hongtao Zhao, Jiaqi Jin, Jiheng Zhang, Nan Wang, Jianyang Du, Shaoshan Hu

**Affiliations:** ^1^ Department of Neurosurgery, The Second Affiliated Hospital of Harbin Medical University, Harbin, China; ^2^ Cancer Center, Department of Neurosurgery, Zhejiang Provincial People’s Hospital, Affiliated People’s Hospital, Hangzhou Medical College, Hangzhou, China; ^3^ Department of Neurosurgery, Shandong Provincial Hospital Affiliated to Shandong First Medical University, Jinan, China

**Keywords:** ferroptosis, low-grade glioma, ferroptosis-related prognostic model, prognostic prediction, immune microenvironment, autophagy, hypoxia

## Abstract

**Background:** Although ferroptosis has been validated to play a crucial role in some types of tumors, the influence of ferroptosis-related genes (FRGs) on the immune microenvironment in low-grade glioma (LGG) remains unclear. In this research, we screen the FRGs to assess the prognosis value and immune microenvironment in LGG, to provide reliable diagnosis and treatment evidence for the clinic.

**Methods:** A total of 1,239 patients of LGG samples were selected for subsequent analyses from The Cancer Genome Atlas, Chinese Glioma Genome Atlas, and the Repository of Molecular Brain Neoplasia Data datasets. Univariate Cox regression analysis was used to screen for prognostic FRGs. Consensus clustering was utilized to determine ferroptosis subtypes of LGG patients. Next, the prognostic model was constructed based on differentially expressed FRGs and validation in the validating datasets. The immune microenvironment, biological pathway, and hypoxia score were explored by single-sample gene set enrichment analysis. The potential response of chemotherapy and immune checkpoint blockade therapy was also estimated. In addition, the correlation between the risk score and autophagy-related genes was examined by the Pearson correlation coefficient.

**Results:** A total of three ferroptosis subtypes were identified by consensus clustering for prognostic FRGs which exhibited different outcomes, clinicopathological characteristics, and immune microenvironment. Afterward, a prognostic model that performed great predictive ability based on nine prognostic FRGs has been constructed and validated. Moreover, the prognostic model had the potential to screen the sensitivity to chemotherapy and immunotherapy in LGG patients. Finally, we also found that the prognostic model has a great connection to autophagy and hypoxia.

**Conclusion:** We developed a ferroptosis-related prognostic model which strongly linked to diagnosis, treatment, prognosis, and recurrence of LGG. This study also reveals the connection between ferroptosis and tumor immune microenvironment.

## Introduction

Low-grade glioma (LGG) belongs to WHO grade II and III gliomas (Louis et al., 2016). It approximately accounts for 15% of the primary intracranial malignant tumors (Sanai et al., 2011). LGG is most commonly seen in young adults aged 35–44 years (Ostrom et al., 2016). At present, the standard therapeutic schedules including surgical resection, adjuvant radiotherapy, and chemotherapy are mainly adopted, but the outcomes are always unfavorable (Semmel et al., 2018). Cancers with same origins, pathologic stages, and clinical stages may have different molecular characterizations (Friedman et al., 2015). Recent studies have identified that molecular pathogenesis is closely related to LGG progression, suggesting the promising prospect of targeted therapy (Bready and Placantonakis, 2019). Consequently, exploring the potential molecular mechanisms will benefit the outcomes of patients with LGG.

Ferroptosis was initially described as a regulated cell death unlike other forms of cell death, in 2012 (Dixon et al., 2012). It is characterized by dysbalance in the regulation of intracellular iron metabolism and membrane lipid peroxidation (Ursini and Maiorino, 2020). The function of ferroptosis in several types of cancer has been reported previously, including breast cancer (Li H. et al., 2022), hepatocellular carcinoma (Chen et al., 2022), gastric cancer (Zhang et al., 2021), head neck squamous cell carcinoma (Lu et al., 2021), lung cancer (Li and Liu, 2022), renal cell carcinoma (Du et al., 2021), ovarian cancer (Li H.-W. et al., 2022), and pancreatic cancer (Liu et al., 2021). Mou et al. (2022) has found that for LGG the SAT1 activation is closely related to ferroptosis upon ROS induction. Based on the sequencing technology, many ferroptosis-related gene (FRG) risk signatures have been developed in LGG to predict prognosis and treatment efficacy (Xu et al., 2021; Zhao et al., 2021; Zheng et al., 2021). However, the influence of FRGs on the tumor microenvironment (TME) in LGG has not been elucidated yet.

In this study, through the screened FRGs from The Cancer Genome Atlas (TCGA), Chinese Glioma Genome Atlas (CGGA), and Repository of Molecular Brain Neoplasia Data (Rembrandt) datasets, a total of 1,239 patients of LGG samples were selected for subsequent analyses. Ferroptosis subtypes with distinct prognosis, immune microenvironment, and clinicopathological and biological processes were identified by consensus clustering. Subsequently, we built the ferroptosis-related prognostic model to quantify the differences between individuals. Beyond that, we also explored the connection between ferroptosis and hypoxia as well as autophagy. Overall, our findings may contribute to the clinical therapeutic strategies for LGG patients.

## Methods

### Dataset Acquisition

The flow chart of this study is shown in Supplementary Figure S1. The mRNA expression profiles with corresponding clinical data of LGG samples were downloaded from TCGA (https://portal.gdc.cancer.gov/repository), CGGA (http://www.cgga.org.cn), and Rembrandt (http://gliovis.bioinfo.cnio.es) datasets. Then, patients with incomplete survival data and histopathological diagnosis were excluded. Ultimately, a total of 1,239 patients of LGG samples were selected for the subsequent analysis. TCGA dataset (*n* = 508) served as the training set. The CGGA (*n* = 592) and the Rembrandt datasets (*n* = 139) were chosen as the validation sets. The available clinical information about the patients is summarized in Supplementary Table S2.

### Identification of Prognostic FRGs and Functional Analysis

The FRGs were obtained from the FerrDb online database (http://www.zhounan.org/ferrdb) (Zhou and Bao, 2020). After merging with LGG transcripts of three cohorts, the univariate Cox regression analysis was used to screen for prognostic FRGs from TCGA, CGGA, and Rembrandt datasets (Supplementary Tables S3–S5). Gene Ontology (GO) and Kyoto Encyclopedia of Genes and Genomes (KEGG) analyses of the intersecting prognostic FRGs were performed using Metascape (https://metascape.org/gp/index.html#/main/step1) (Zhou et al., 2019).

### Classification of Molecular Subtypes by Consistent Clustering

Based on the intersecting prognostic FRGs, the ‘ConsensusClusterPlus’ package in R was utilized for the consistent clustering to determine ferroptosis subtypes of LGG patients from TCGA, CGGA, and Rembrandt datasets. The k-value (ranging from 2– 9) was used for determining the best cluster number. The overall survival (OS) analysis among different clusters was calculated using the Kaplan–Meier method.

### Evaluation of Immune Infiltration in the TME

The immune-stromal component of the TME for each sample was calculated with the ESTIMATE algorithm (Yoshihara et al., 2013), which is commonly represented as three kinds of scores named ImmuneScore, StromalScore, and ESTIMATEScore. Using the single-sample gene set enrichment analysis (ssGSEA), the relative infiltration of 28 immune cells in TME and the activity levels of typical biological pathways in individual samples were calculated.

The anti-tumor immune response can be described as a sequence of gradual procedures, including the release of cancer cell antigens (Step 1), cancer antigen presentation (Step 2), priming and activation (Step 3), trafficking of immune cells to tumors (Step 4), infiltration of immune cells into tumors (Step 5), recognition of cancer cells by T cells (Step 6), and killing of cancer cells (Step 7). In this research, we explored the connection between ferroptosis subtypes and anti-tumor immune response. The anti-tumor activity score of each sample in TCGA was obtained from Tumor Immunophenotype Profiling (TIP, http://biocc.hrbmu.edu.cn/TIP/index.jsp) (Xu et al., 2018).

### Mutational Signature Analyses

The tumor mutation burden (TMB) was defined as the total number of somatic mutations per megabase in tumor tissue. Much like the immunosuppressive microenvironment, TMB is also critical in anti-tumor immunotherapy. Therefore, we calculated the TMB of each sample in LGG based on TCGA mutation data. The R package ‘maftools’ was used to process and present the mutation data (Mayakonda et al., 2018).

### Identification and Validation of the Prognostic Model

According to the intersecting prognostic FRGs, we identified the differentially expressed FRGs (DE-FRGs) with adjusted *p*-value < 0.05 between cluster-1 and cluster-3 using the ‘limma’ package in R. We further applied the ‘glmnet’ package in R to perform the least absolute shrinkage and selection operator (LASSO) regression analysis for narrowing the range of genes those were upregulated in DE-FRGs. Then, the risk score for each sample can be calculated using the following formula.

$$riskScore=\overset{n}{\underset{i=1}{\sum }}Coef\left(X_{i}\right)*\;Exp\left(X_{i}\right).$$

In the formula, *Coef (X*_*i*_ *)* represents the coefficient of each FRG, and *Exp (X*_*i*_ *)* stands for the gene expression levels of those FRGs. The patients were divided into low- and high-risk groups according to the median risk score. The risk score of patients from CGGA and Rembrandt datasets can also be calculated to validate the efficacy of the prognostic model.

The Kaplan–Meier method was used to draw survival curves. Meanwhile, the area under the curves (AUCs) of receiver operating characteristic (ROC) curves was calculated to evaluate the predictive ability of 1, 3, and 5 years of survival. To explore whether the prognostic model could be used as an independent factor of OS in LGG, univariate and multivariate Cox regression analyses were performed. Next, combining all independent prognostic factors from the previous step, the nomogram was built using the R package ‘rms’. The calibration curve was used to evaluate the accuracy of the nomogram.

### Prediction of Chemotherapeutic and Immune Checkpoint Blockade Therapy Response

Temozolomide is the most commonly used chemotherapeutic in LGG therapy. Therefore, the chemotherapeutic response of temozolomide for each patient was predicted by the Genomics of Drug Sensitivity in Cancer (https://www.cancerrxgene.org/). The prediction of half-maximal inhibitory concentration (IC_50_) values was conducted using the R package ‘pRRophetic’ (Geeleher et al., 2014).

Tumor immune dysfunction and exclusion (TIDE, http://tide.dfci.harvard.edu/) is a calculation method based on the induction of T-cell dysfunction in tumors with high infiltration of cytotoxic T lymphocytes (CTLs) and the prevention of T-cell infiltration in tumors with low CTL levels (Jiang et al., 2018). The subclass mapping method (SubMap, https://www.genepattern.org/) is an unsupervised algorithm that reveals common subtypes between independent datasets (Hoshida et al., 2007). In this study, the TIDE and SubMap algorithms were used to estimate the immune checkpoint blockade (ICB) therapy response of LGG patients.

### Gene Set Enrichment Analysis

Gene set enrichment analysis (GSEA) was performed using GSEA software (v4.0.0) to identify signaling pathways regulated by the prognostic model. The hallmark gene set collection is provided by the Molecular Signatures Database (MSigDB) (http://www.broad.mit.edu/gsea, v7.4). Gene sets with | NES | > 1 and nominal *p*-value < 0.05 were considered significant.

### Correlations of the Prognostic Model With the Autophagy and Hypoxia Score

We retrieved the autophagy-related genes (ARGs) from the Human Autophagy Database (http://www.autophagy.lu/autophagy.html). The relationship between ARGs and the risk score was estimated by the Pearson correlation coefficient.

The hypoxia-related gene set was retrieved from the MSigDB. To obtain hypoxia scores, the enrichment fraction of the hypoxia pathway in each sample was quantified by the ssGSEA algorithm.

### Statistical Analysis

R software (v3.6.0) and GraphPad Prism (v9.3.1) were used for statistical analyses and visualization. The survival differences of Kaplan–Meier analysis were assessed with the log-rank test through the ‘survminer’ package in R. Differences among the inter-group were compared using the Wilcox test. The value of *p* < 0.05 was considered statistically significant.

## Results

### Identification of the Prognosis-Related FRGs in LGG

**FIGURE 1:**
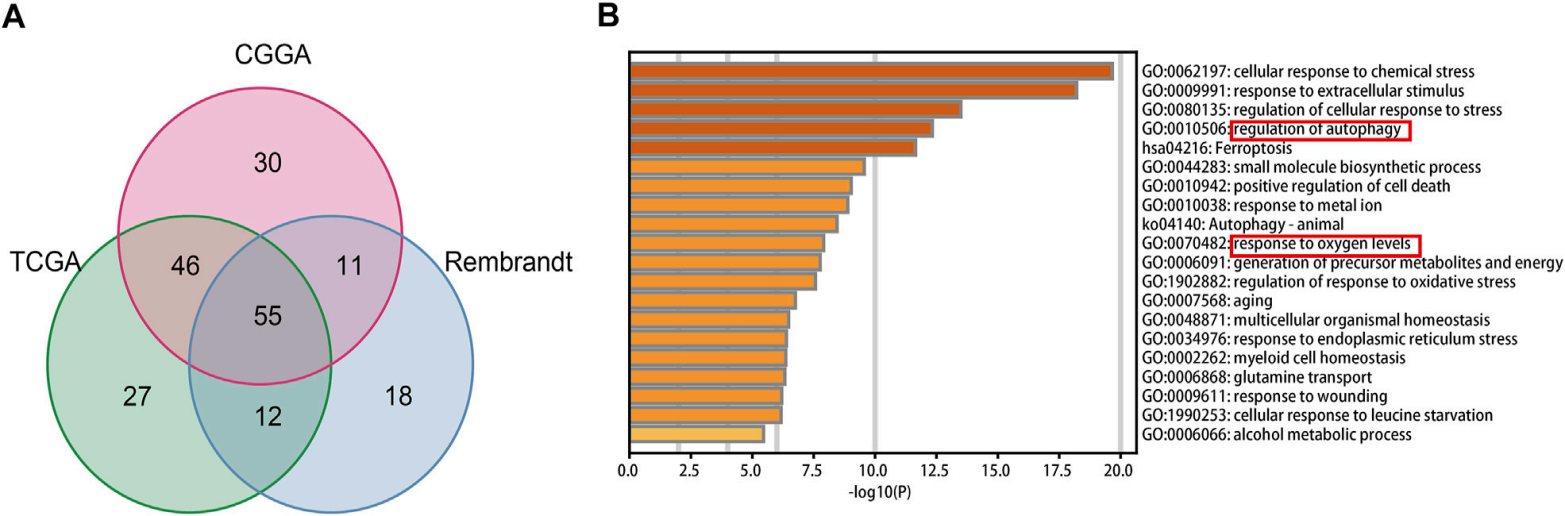
Identification and functional enrichment analyses of the intersecting FRGs in TCGA, CGGA, and Rembrandt datasets. **(A)** Venn diagram to identify the intersecting FRGs from TCGA, CGGA, and Rembrandt datasets. **(B)** GO and KEGG analyses of the intersecting FRGs.

In total, fifty-five intersecting prognostic FRGs were identified by univariate Cox regression analysis in TCGA, CGGA, and Rembrandt databases (Figure 1A, Supplementary Table S6). After that, we performed a functional analysis using Metascape Online. As shown in Figure 1B, the GO analysis results suggest that the intersecting prognostic FRGs are enriched in response to the regulation of autophagy, metal ion, and oxygen levels. The KEGG pathway analysis revealed that the intersecting prognostic FRGs were enriched in ferroptosis and autophagy signaling pathways.

### Consensus Clustering Determined Ferroptosis-Related Clusters of LGG

**FIGURE 2:**
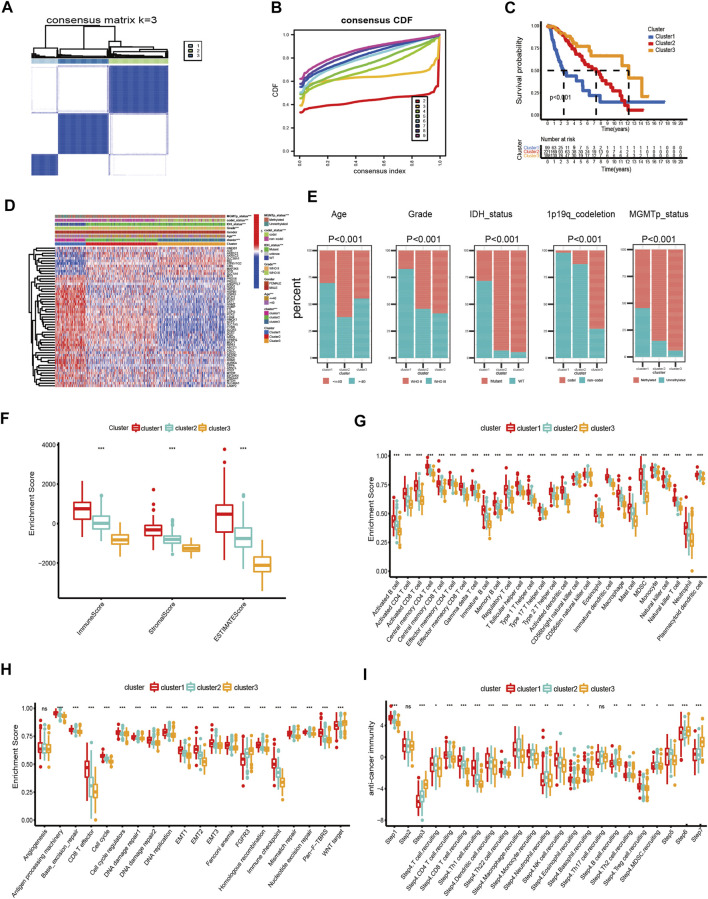
Differential clinicopathological features and immune landscape of LGG among different clusters in TCGA dataset. **(A)** Consensus clustering matrix for *k* = 3. **(B)** Cumulative distribution function curves for *k* = 2–9. **(C)** Kaplan–Meier curve of overall survival among three clusters. **(D)** Heatmap and clinicopathological features of the three clusters. **(E)** Proportion of clinical characteristics in three clusters. **(F–I)** Distribution of ImmuneScore **(F)**, 28 immune cells **(G)**, typical biological pathways **(H)**, and activity score of the anti-tumor immune response **(I)** across the three ferroptosis subtypes. The horizontal line of the box plot represents the median values (*p < 0.05, **p < 0.01, ***p < 0.001; ns, non-significant).

In this study, we explored the expression levels of fifty-five intersecting prognostic FRGs to construct consensus clusters. The ‘ConsensusClusterPlus’ package of R was exploited to confirm the ideal cluster numbers by calculating the average cluster consistency and intercluster coefficient variation of each class number. Ultimately, the consensus matrixes (Figure 2A) and cumulative distribution function (CDF) curves (Figure 2B) showed that *k* = 3 was the stable clustering number of FRGs. The LGG patients in TCGA dataset were divided into three groups named cluster-1 (*n* = 99), cluster-2 (*n* = 221), and cluster-3 (*n* = 188). Compared with cluster-2 and cluster-3, the Kaplan–Meier survival plot showed that patients in cluster-1 had the worst prognosis (Figure 2C). In addition, we also got three ferroptosis subtypes in CGGA and Rembrandt databases (Supplementary Figures S2A,B, S3A,B). Similar to TCGA dataset, the survival analysis also showed significant differences among different subtypes in the validating datasets (Supplementary Figures S2C, S3C). Meanwhile, the heatmap showed clinical and molecular features and different expression levels of fifty-five intersecting prognosis FRGs among different clusters in three datasets (Supplementary Figures 2D, S2D, S3D). Next, we further analyzed the distribution of various clinical features in different subgroups of three datasets (Figure 2E). In TCGA dataset, patients in cluster-1 have a higher proportion of age >40, WHO III, isocitrate dehydrogenase (IDH) wild type, O6-methylguanine-DNA methyltransferase promoter (MGMTp) unmethylated, and 1p19q non-codeletion which corresponds to the poor prognosis, whereas the cluster-3 patients are lower in these aforementioned features. Similar to TCGA dataset, the clinical traits also present marked differences among different clusters in the CGGA and Rembrandt datasets (Supplementary Figures S2E, S3E).

### The Immune Microenvironment and Mutational Status in the Subtypes of Ferroptosis

First, we analyzed the immune scores of the three ferroptosis subtypes and found that the ImmuneScore, StromalScore, and ESTIMATEScore were higher in cluster-1 than those of the other two subtypes (Figure 2F). Subsequently, a correlation between the immune cell composition and ferroptosis subtypes was explored. As shown in Figure 2G, cluster-1 has a higher score of immune cells, followed by cluster-2 and cluster-3. We further analyzed the enrichment of typical biological processes in different clusters. The result revealed that cluster-1 was remarkably enriched in most oncogenic pathways (Figure 2H). The aforementioned results were also validated in two validation datasets (Supplementary Figures S2F–H, S3F–H).

T cell plays a vital role in anti-tumor immunotherapy. We analyzed the correlations between the ferroptosis subtypes and the activities of the anti-tumor immune response (Figure 2I). The anti-tumor activity score of the release of cancer cell antigens (Step 1) and a large proportion of immune cell recruiting (Step 4) were significantly higher in cluster-1. The steps of priming and activation (Step 3), recognition of cancer cells by T cells (Step 6), and killing of cancer cells (Step 7) were higher in cluster-3. However, cluster-2 has a higher score in the fraction of immune cell recruiting (Step 4) and infiltration of immune cells into tumors (Step 5). In addition, there is no significant difference in cancer antigen presentation (Step 2) among the three subgroups.

**FIGURE 3:**
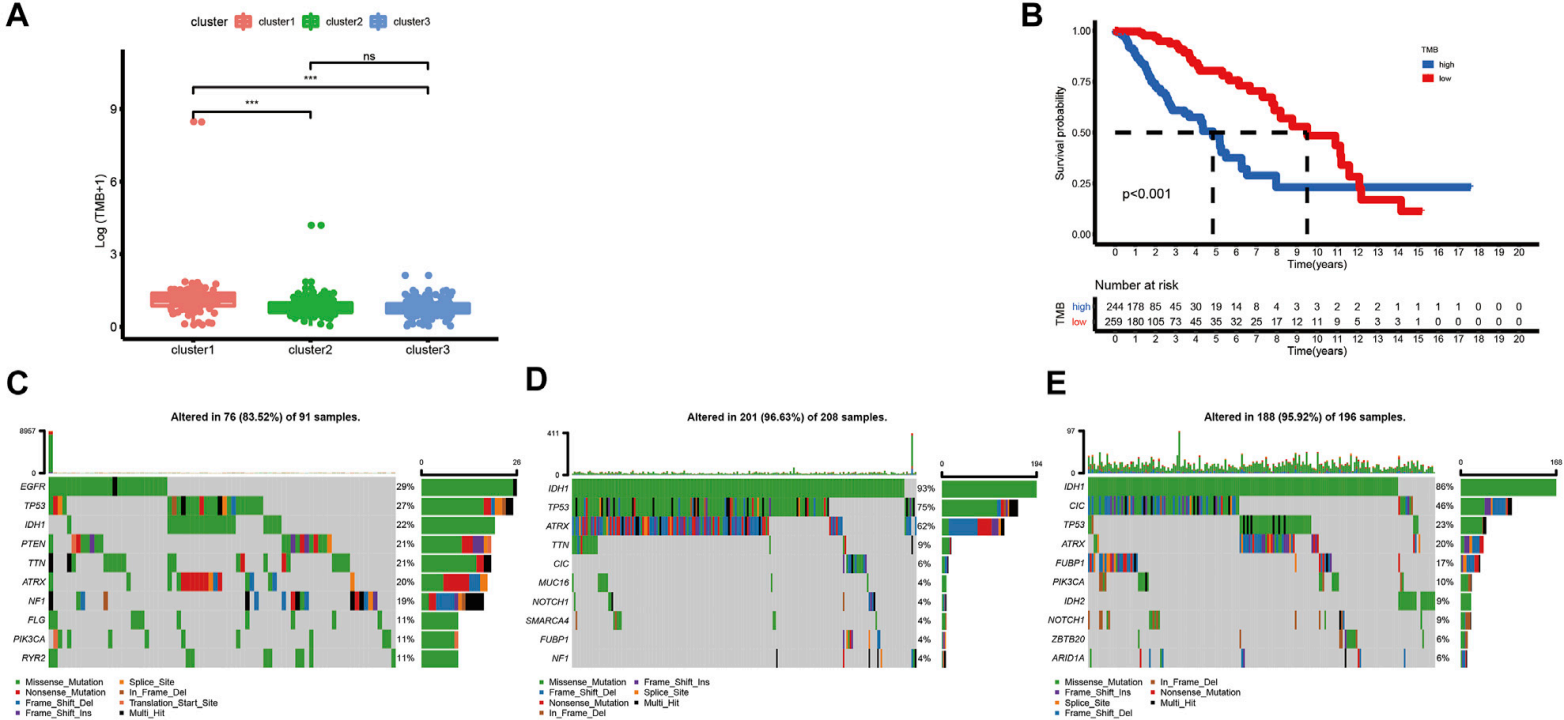
Correlations between the ferroptosis subtypes and somatic variants in TCGA dataset. **(A)** Differences in TMB levels among different ferroptosis subtypes. **(B)** Correlation between TMB and prognosis in LGG patients. **(C-E)** Distribution of the top ten variants of mutated genes among different ferroptosis subtypes. The horizontal line of the box plot represented the median values (*p < 0.05, **p < 0.01, ***p < 0.001; ns, non-significant).

Although high TMB is closely associated with a poor prognosis of glioma, it is also associated with better responses to immunotherapy (Bouffet et al., 2016; Touat et al., 2020; Yin et al., 2020). In this research, we calculated TMB scores for each sample with mutations in TCGA database to compare the differences between various clusters. The TMB level of cluster-1 is higher than that of others. However, there is no significant difference between cluster-2 and cluster-3 (Figure 3A). Meanwhile, we found that there is a significant correlation between TMB and prognosis in LGG patients (Figure 3B). Furthermore, among these three ferroptosis subtypes, cluster-2 had the highest mutation rate (96.63%), followed by cluster-3 (95.92%) and cluster-1 (83.52%). Previous studies have reported that IDH1 and IDH2 mutations were closely related to the prognosis of glioma patients (Yan et al., 2009). In this study, we noticed that the IDH mutation in cluster-3 (86% IDH1 and 9% IDH2) is higher than that in cluster-1 (22% IDH1) and cluster-2 (93% IDH1), indicating the vital role of IDH mutation in LGG patients (Figures 3C–E).

### Construction and Validation of the Prognostic Model Based on FRGs

**FIGURE 4:**
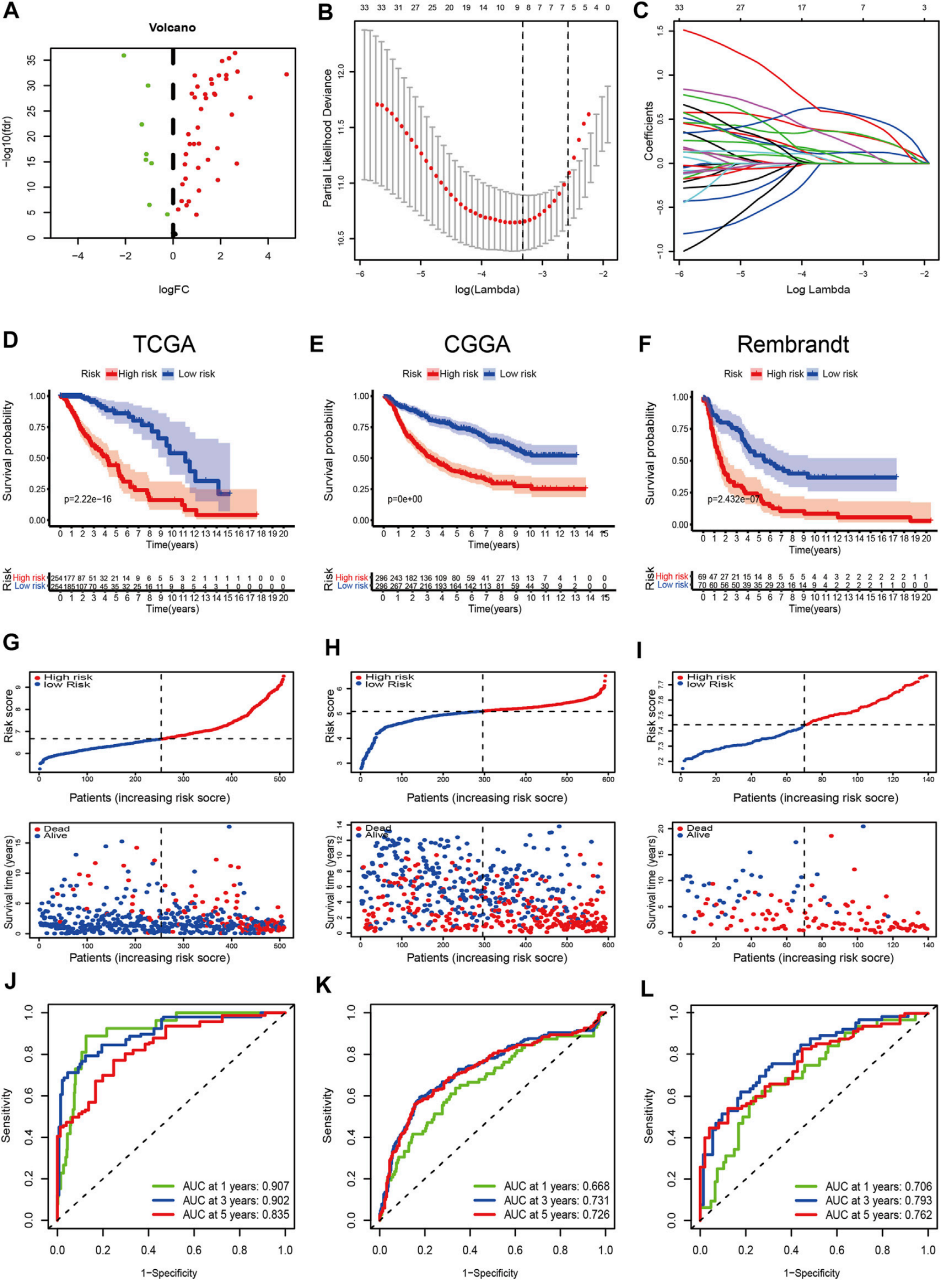
Construction and validation of the prognostic model. **(A)** Volcano plot of DEGs between cluster-1 and cluster-3; red indicates downregulated genes, and green indicates upregulated genes. **(B)** LASSO coefficient profiles of the 41 upregulated DE-FRGs in TCGA dataset. **(C)** Cross-validation for tuning the parameter selection in the LASSO analysis. **(D–F)** Kaplan–Meier curves for survival in the TCGA, CGGA, and Rembrandt datasets. **(G–I)** Distribution plots of the risk score and survival status in the TCGA, CGGA, and Rembrandt datasets. **(J–L)** ROC curve analyses for predicting 1-, 3-, and 5-year OS in the TCGA, CGGA, and Rembrandt datasets.

**FIGURE 5:**
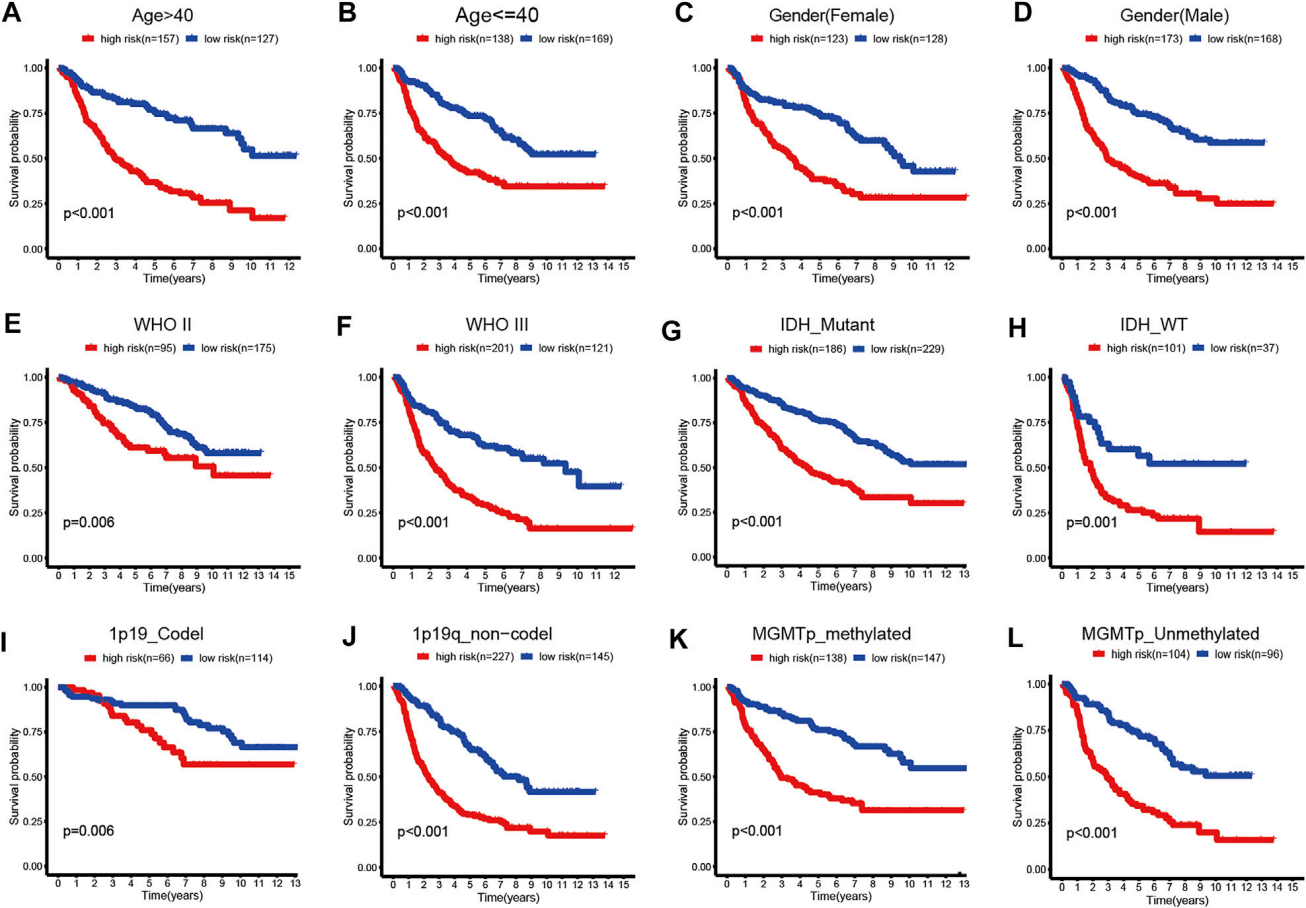
Kaplan–Meier survival curves for the low- and high-risk groups stratified by clinicopathological variables in TCGA dataset. **(A,B)** Age. **(C,D)** Gender. **(E,F)** WHO grade. **(G,H)** IDH_status. **(I,J)** 1p19q_status. **(K,L)** MGMTp_status.

There were 49 DE-FRGs obtained from cluster-1 and cluster-3, including 41 upregulated, and 8 downregulated (Figure 4A). Taking advantage of the upregulated DE-FRGs, we constructed a new ferroptosis-related prognostic model according to the LASSO regression with the optimal lambda value (Figures 4B,C). The calculated coefficient of the nine FRGs is shown in Supplementary Table S7. According to the median cut-off value, the model categorized the patients into low- and high-risk groups. The Kaplan–Meier survival curve in TCGA databases indicated that patients in the high-risk group were associated with worse outcomes (Figure 4D). The distribution plot of the risk score and survival status showed that the risk score was strongly positively correlated with the death of LGG patients (Figure 4G). The AUC values of the prognostic model for FRGs were 0.907 (1-year), 0.902 (2-year), and 0.835 (5-year), which exhibited a remarkable predictive performance (Figure 5J).

We validated the applicability of the prognostic model in the CGGA and Rembrandt datasets. Similarly, patients in the low-risk group had better prognosis (Figures 4E,F,H,I). The AUC values for 1-year, 2-year, and 5-year survival in the CGGA dataset were 0.688, 0.731, and 0.726, respectively (Figure 4K). The AUC values in the Rembrandt dataset were 0.706, 0.793, and 0.762, respectively (Figure 4L). All the results prove that our prognostic model reveals favorable specificity and sensitivity.

In addition, we performed subgroup analyses according to age, gender, grade, IDH_status, 1p19q_status, and MGMTp_status in CGGA and TCGA datasets. Patients in the high-risk group were predicted with a worse prognosis in all subgroups (Figures 5A–L, and Supplementary Figures S4A–L). It is prompted that the prognostics model is better clinically applicable.

### Construction and Validation of a Prognostic Nomogram

**FIGURE 6:**
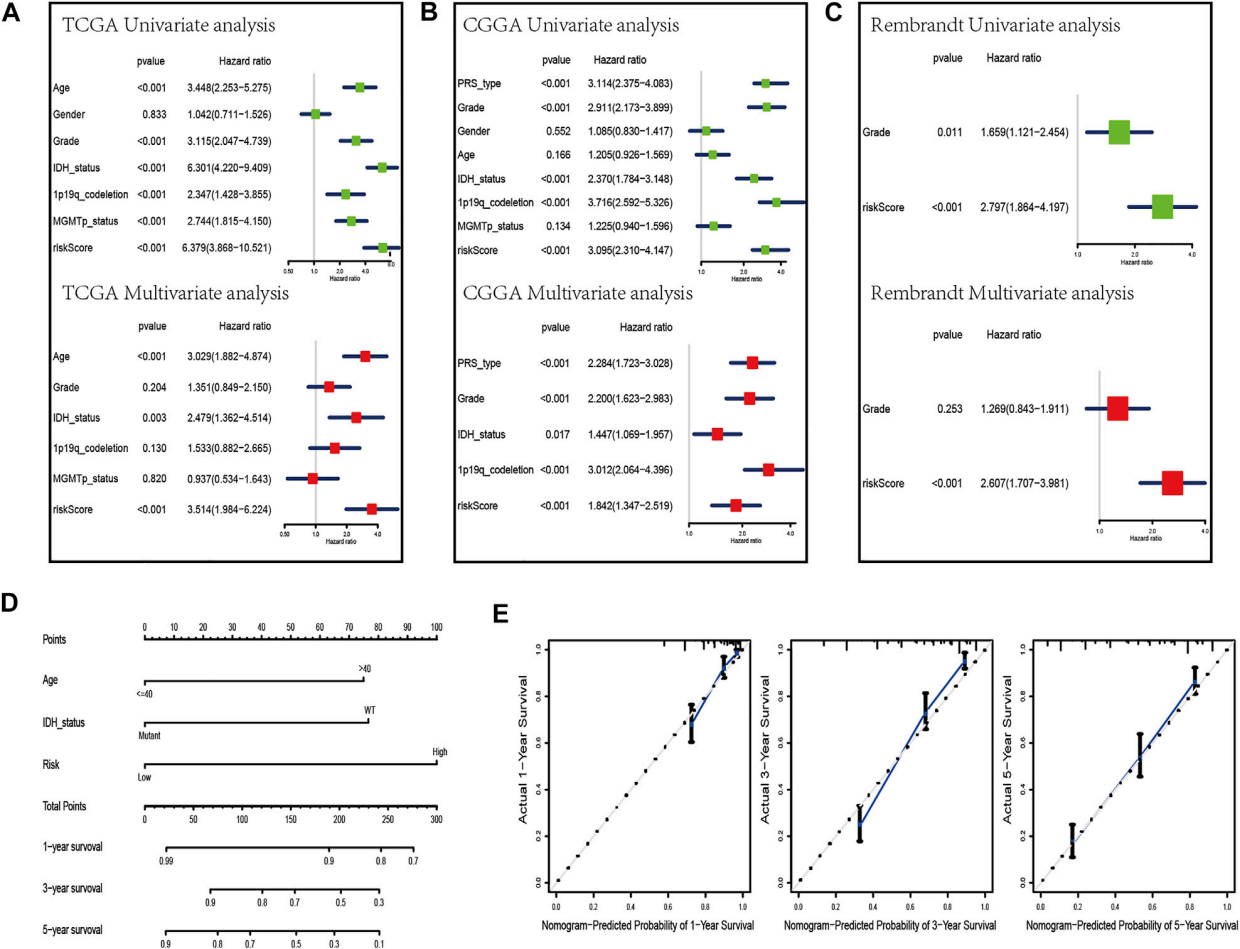
Development of a nomogram by integrating the risk score and clinicopathological characters in the TCGA cohort. **(A–C)** Univariate analysis and multivariate analysis containing risk score and clinical factors in TCGA **(A)**, CGGA **(B)**, and Rembrandt **(C)** datasets. **(D)** Nomogram constructed to predict OS rates at 1, 3, and 5 years. **(E)** Calibration curves predicted 1-, 3-, and 5-year survival.

To evaluate whether the risk scores can be applied as an independent prognostic biomarker, univariate and multivariate Cox regression analyses were performed in TCGA, CGGA, and Rembrandt datasets (Figures 6A–C). The results suggest that the risk score was always an independent prognostic factor in both univariate and multivariate Cox regression analyses. Meanwhile, we constructed a nomogram with the independent prognostic parameters for the OS in TCGA and CGGA datasets (Figures 6D, Supplementary Figure S5A). Meanwhile, the calibration plot showed that the predicted power was similar to the actual observations (Figures 6E, Supplementary Figure S5B).

### The Immune Microenvironment and Mutational Status in Distinct Risk Groups

**FIGURE 7:**
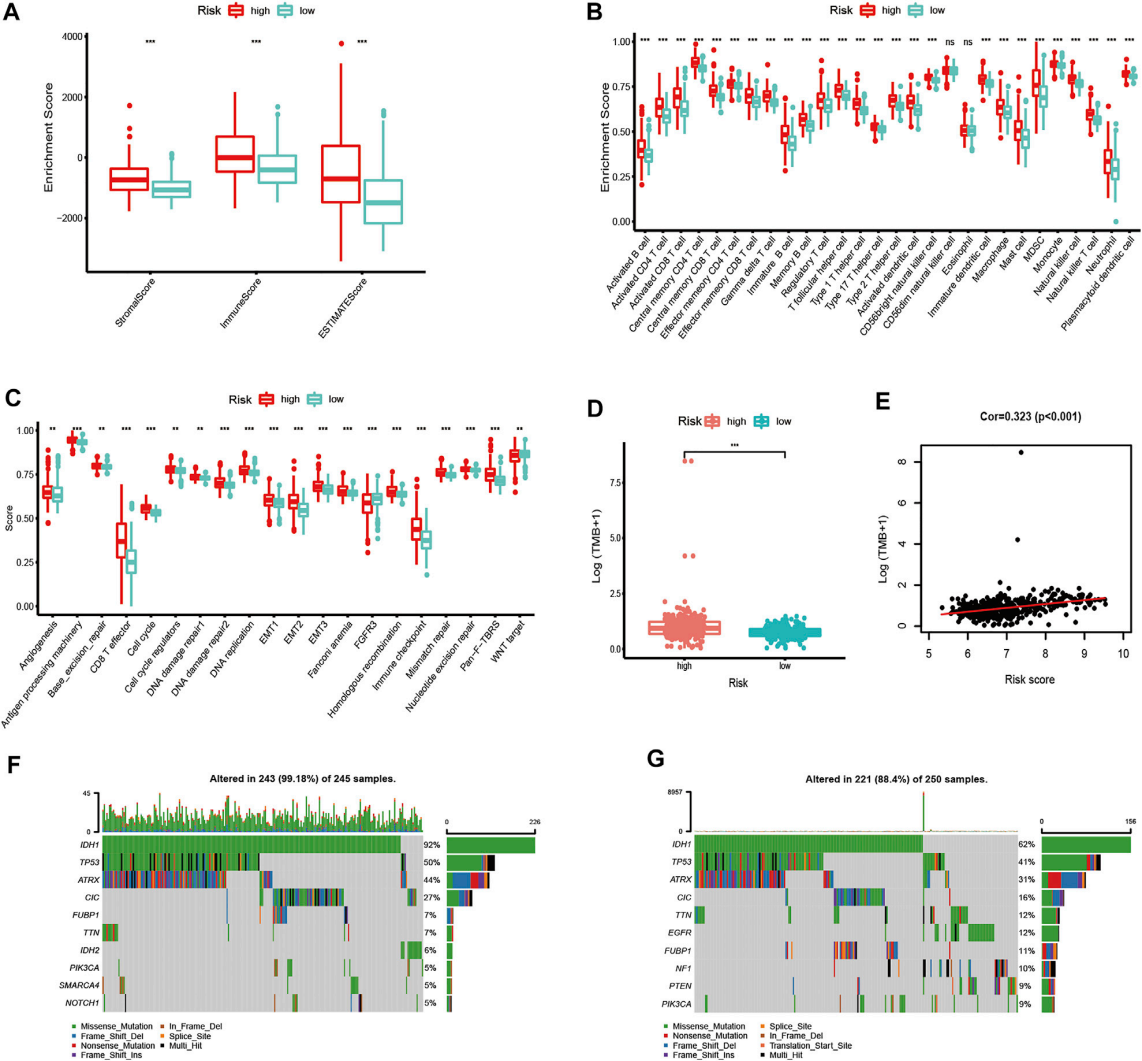
Immune microenvironment, biological process characteristics, and prediction of chemotherapeutic response among distinct risk groups. **(A–D)** Distribution of ImmuneScore **(A)**, 28 immune cells **(B)**, typical biological pathways **(C)**, and TMB levels **(D)** between the high- and low-risk groups in TCGA dataset. **(E)** Correlation between TMB and risk score in LGG patients. **(F,G)** Mutation rates of top 10 mutated genes in low- and high-risk groups. The horizontal line of the box plot represents the median values (*p < 0.05, **p < 0.01, ***p < 0.001; ns, non-significant).

We further evaluated the difference in immune scores, immune cell composition, and typical biological processes between the two risk groups in TCGA dataset. As the results reflected, the immune scores and the majority of the immune cell composition were higher in the high-risk group (Figures 7A,B). Meanwhile, it is not surprising that most oncogenic pathways were enriched in the high-risk group compared with the low-risk group (Figure 7C). The same results were confirmed in the CGGA and the Rembrandt datasets (Supplementary Figures S6A–F).

We next analyzed the differences in somatic mutation among the two risk groups in TCGA dataset. As shown in Figure 7D, there was a significant difference in TMB levels between the high- and low-risk groups. The risk score was positively correlated to the level of the TMB (Figure 7E). Moreover, patients in the high-risk group have a higher mutation rate than those in the low-risk group (Figures 7F,G).

### Sensitivity to Chemotherapies and ICB Therapy in Distinct Risk Groups

**FIGURE 8:**
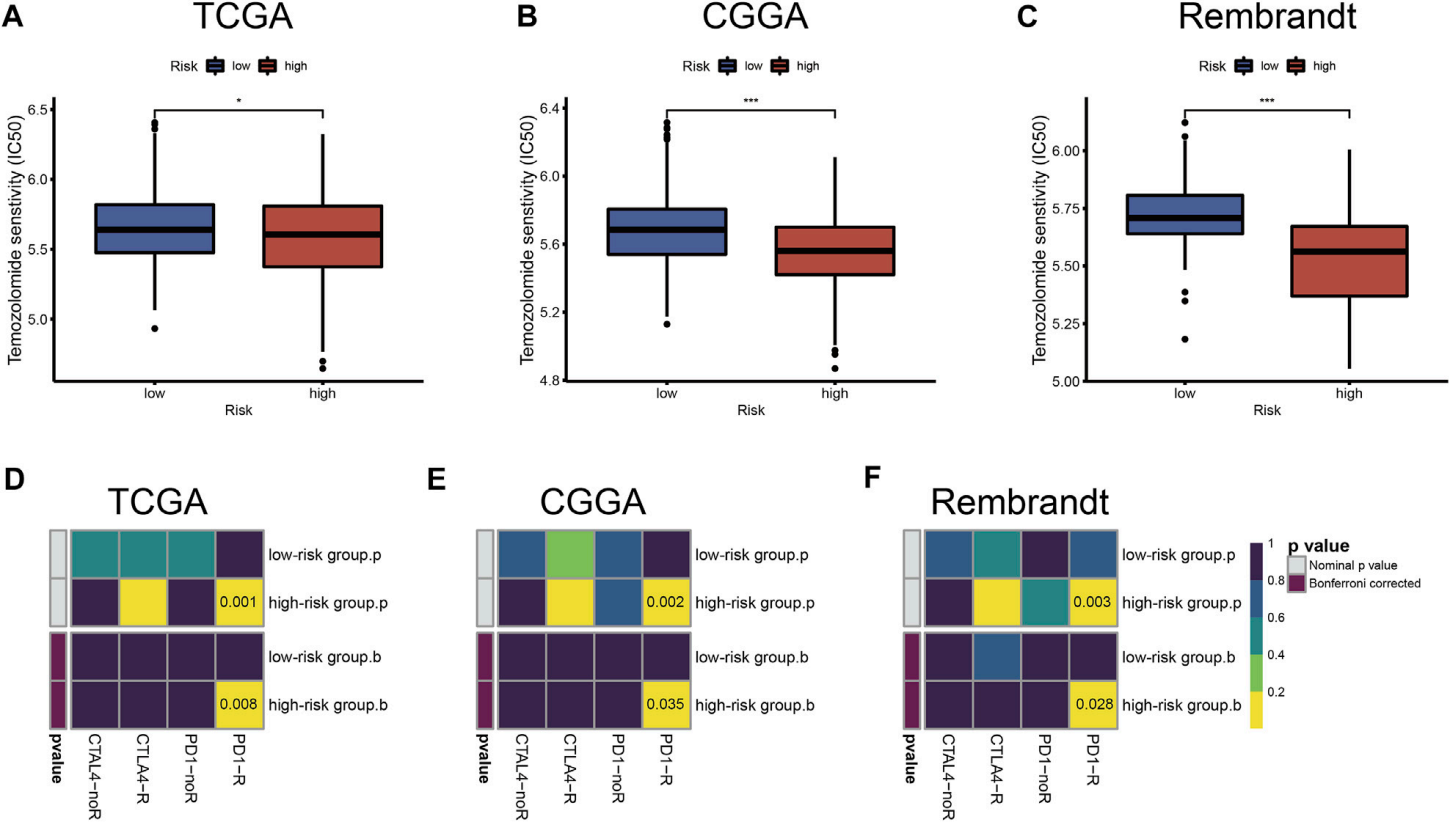
Chemotherapeutics and ICB therapy responses in high- and low-risk groups with LGG. Drug sensitivity of temozolomide in TCGA **(A)**, CGGA **(B)**, and Rembrandt **(C)** datasets. ICB therapy responses to anti-PD1 and anti-CTLA4 treatments of LGG in TCGA **(D)**, CGGA **(E)**, and Rembrandt **(F)** datasets. The horizontal line of the box plot represents the median values (*p < 0.05, **p < 0.01, ***p < 0.001; ns, non-significant).

To further study and characterize drug responses of temozolomide in LGG patients, we assessed differences in drug sensitivity between the high- and low-risk groups by analyzing the IC_50_ of temozolomide. We found that in TCGA, CGGA, and Rembrandt datasets, patients in the high-risk group were more sensitive to temozolomide (Figures 8A–C).

Immune checkpoint blockades that target CTLA-4 and PD-1/PD-L1 have shown some promise against glioma (Saha et al., 2017; Zhao et al., 2019), but only a fraction of patients respond to treatment. According to the TIDE and SubMap algorithms, the expression profiles of TCGA, CGGA, and Rembrandt datasets were compared with a published dataset containing 47 melanoma patients who responded to immunotherapy. We found that treatment with PD-1 showed better results in the high-risk group (Bonferroni correction *p* < 0.05) (Figures 8D–F).

### Functional Analysis of the Prognostic Model

**FIGURE 9:**
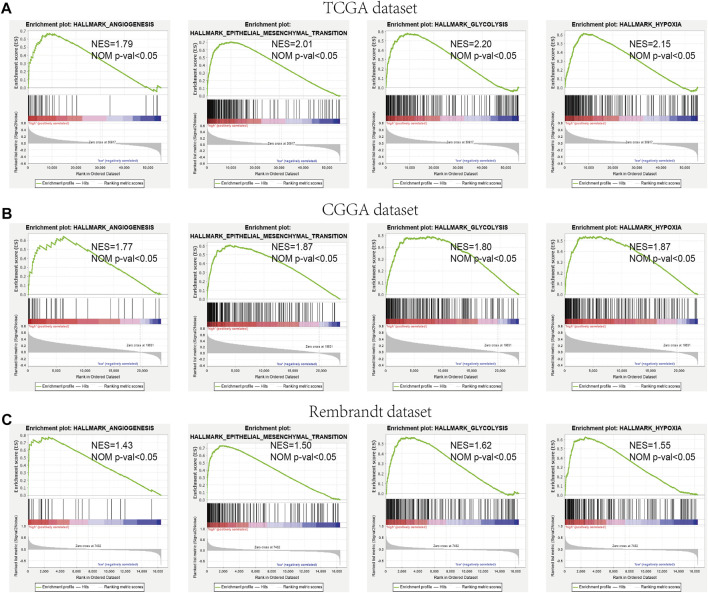
GSEA comparisons of the low- and high-risk groups. **(A–C)** Common functional gene sets enriched in the high-risk group compared to the low-risk group in TCGA **(A)**, CGGA **(B)**, and Rembrandt **(C)** datasets.

We further verified the different functional phenotypes involved in the high- and low-risk groups *via* GSEA. The finding disclosed that several pathways, such as angiogenesis, epithelial–mesenchymal transition, hypoxia, and glycolysis, were significantly activated in the high-risk group (Figure 9A). These findings were further validated in the CGGA and Rembrandt datasets (Figures 9B,C).

### Identification of the Hypoxia Correlation With the Ferroptosis-Related Prognostic Model

**FIGURE 10:**
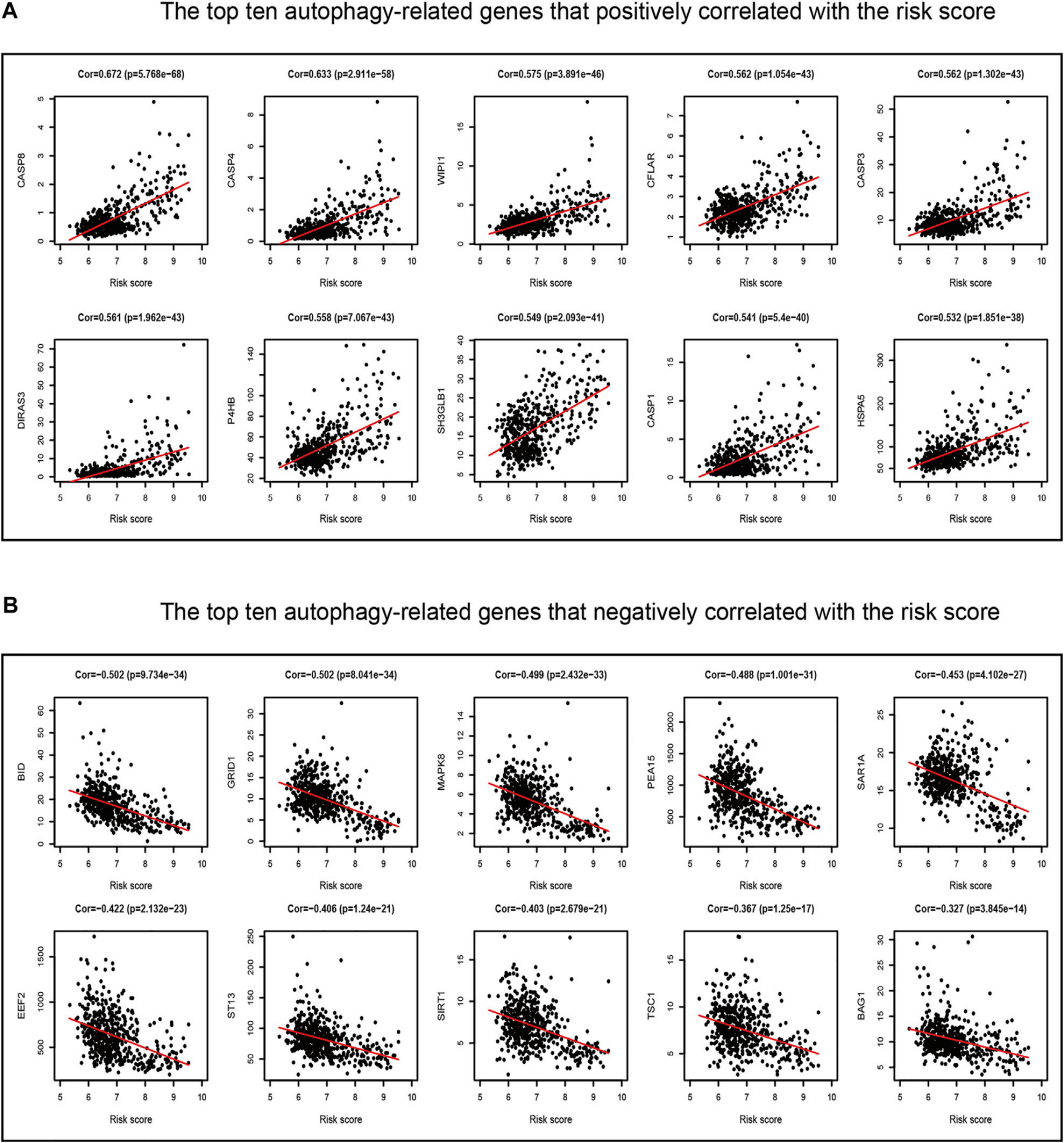
Correlations between the ferroptosis-related prognostic model and ARGs in TCGA dataset. **(A)** Top ten ARGs positively correlated with the risk score. **(B)** Top ten ARGs negatively correlated with the risk score.

Our previous study found that the intersecting FRGs were correlated to biological processes of oxygen metabolism through GO and KEGG analyses. First, the Pearson correlation coefficient was taken advantage of evaluating the connection between ARGs and the risk score of the prognostic model. Among the 210 ARGs, a total of 175 (83.3%) ARGs were significantly correlated with risk scores, of which 126 were positively correlated, and 49 were negatively correlated (Supplementary Table S8). The top 10 ARGs positively correlated (*CASP8, CASP4, WIPI1, CASP3, CFLAR, DIRAS3, P4HB, SH3GLB1, CASP1, and HSPA5*) with the risk score and the top 10 negative relationships (*BID, GRID1, MAPK8, PEA15, SAR1A, EEF2, ST13, SIRT1, TSC1, and BAG1*) with the risk score are shown in Figures 10A, B.

**FIGURE 11:**
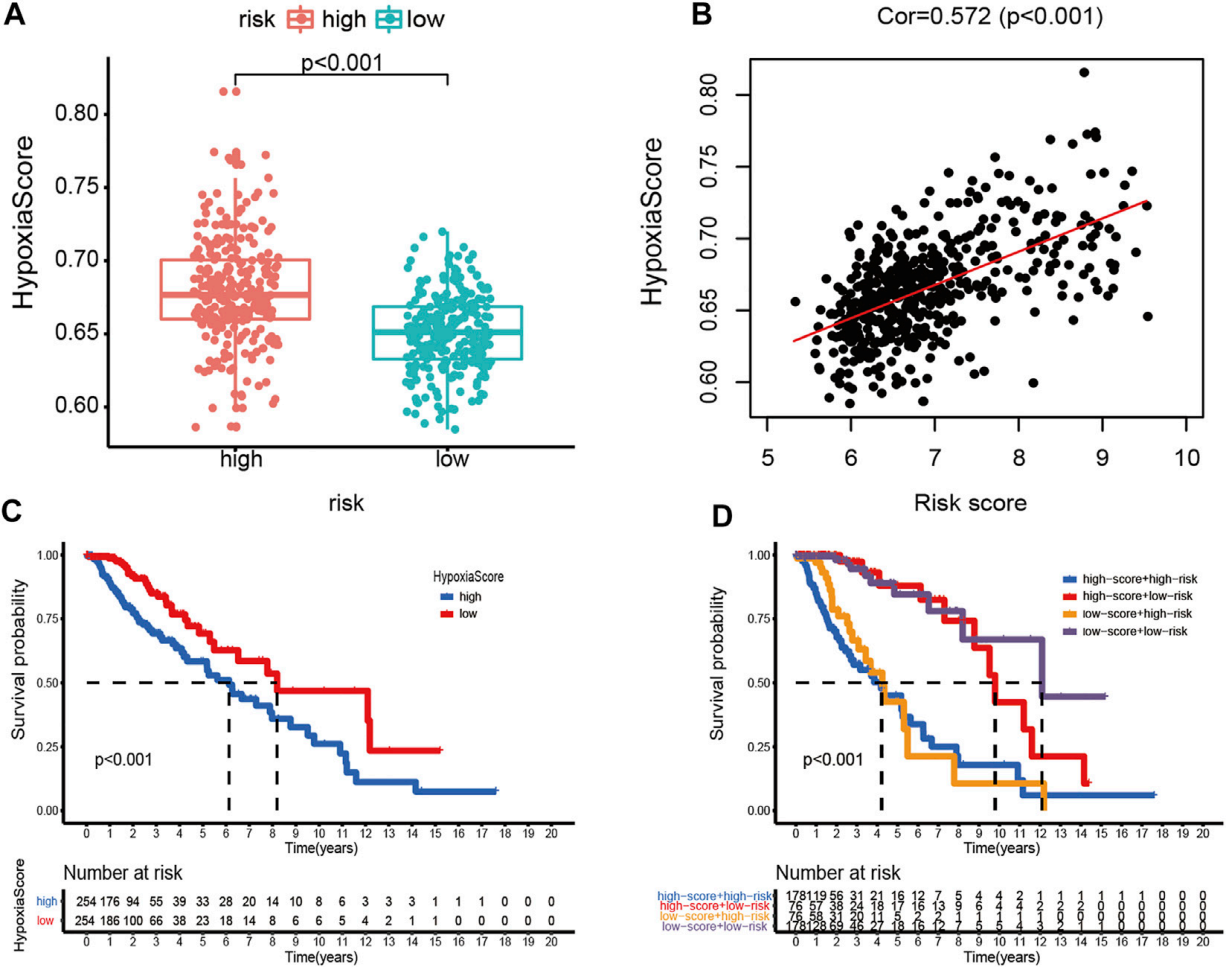
Hypoxia score analysis. **(A)** Hypoxia scores between high- and low-risk groups. **(B)** Correlation between the hypoxia score and risk score in LGG patients. **(C)** Kaplan-Meier survival curves of OC patients with high or low hypoxia score. **(D)** Kaplan-Meier survival curves of four subgroups based on the risk score and hypoxia score.

Afterward, the hypoxia enrichment score of each patient was calculated. As shown in Figure 11A, patients in the high-risk group had a higher hypoxia score. The risk score was positively correlated to the hypoxia score (Figure 11B). Afterward, the patients were divided into two groups by the median of hypoxia scores. The Kaplan-Meier survival curves performed an unfavorable prognosis of the high hypoxia score patients (Figure 11C). Figure 11D shows that a low-risk score combined with a low hypoxia score group performed better outcomes compared with the other groups.

## Discussion

LGGs are a fatal, invading, and heterogeneous group of tumors and usually results in progressive neurological disability and adverse clinical outcomes (Hayhurst, 2017). Different from necrosis, apoptosis, autophagy, and pyroptosis, ferroptosis is a new type of regulated cell death (Dixon et al., 2015), which is closely related to glioma tumorigenesis, progression, and tumor microenvironment (Xu et al., 2021). Therefore, the effective prognostic biomarkers relying on the FRGs will benefit the clinical treatment of patients with LGG.

The present study commits to detecting the correlation between the ferroptosis subtypes and the ferroptosis-related prognostic model and the immune microenvironment. Ulteriorly, we identified potential biomarkers for prognosis prediction and target therapy. According to the prognostic FRGs, the LGG patients were initially sorted into three ferroptosis states, which exhibited different outcomes, clinicopathological features, immune landscape, and biological processes. Later on, we constructed an FRG-based prognostic model associated with LGG patients. Our results revealed that the prognostic model performed a predictive performance with satisfactory sensitivity and specificity. Meanwhile, the prognosis of LGG was affected by the model which was an independent factor. We also studied the immune microenvironment, chemotherapies, and ICB therapy between different risk groups. All the aforementioned results in the validating datasets were verified. Furthermore, the relationship between the prognostic model and autophagy as well as hypoxia was explored.

The immune microenvironment has a great effect on tumor proliferation and molecular heterogeneity (Barthel et al., 2021). In this study, the immune microenvironment of each cluster in TCGA dataset was first evaluated. We found that the immune score and immune cell infiltration in cluster-1 were higher than those in the others. However, the result of cancer immunity cycles showed that the anti-tumor activities of priming and activation (Step 3), recognition of cancer cells by T cells (Step 6), and killing of cancer cells (Step 7) in cluster-1 are lower. Meanwhile, the biological processes including stromal activation (EMT, Pan−F−TBRS) and immune activation (CD8 T effector, antigen processing machinery, and immune checkpoint) pathways in cluster-1 were higher than those in the other two clusters. Considering that glioma is characterized by a ‘cold’ tumor (Jackson et al., 2019), we deduce that the reason for the patients’ poor outcomes in cluster-1 probably derived from the deficiency of ‘effective T cells’ which affect the immunosuppression microenvironment. We also discovered that cluster-1 with a higher TMB level suggested that patients may gain a positive efficacy from immunotherapy. Although patients in cluster-1 have the highest TMB level, the mutation rate is lower than that in other clusters. Research findings show that IDH mutation has a significant correlation to the prognosis of glioma (Hartmann et al., 2010; Turkalp et al., 2014; Eckel-Passow et al., 2015). In this study, patients in cluster-3 have the highest IDH mutation rate among the three clusters, corresponding to better outcomes. Interestingly, as a potent tumor suppressor, CIC mutation merely occurred in the top 10 mutated genes of cluster-2 (6%) and cluster-3 (46%) (Wong and Yip, 2020). Similarly, PTEN mutation, which results in the loss of tumor-suppressive function in LGG (Endersby and Baker, 2008), exclusively appeared in the top 10 mutated genes of cluster-1.

For the convenience of the calculation of the TME landscapes in individuals, we evaluated the immune microenvironment among the different risk groups. Similar to the aforementioned results, anti-tumor immune responses are both activated and suppressed in the high-risk group. GSEA revealed that the regulation of “angiogenesis,” “epithelial–mesenchymal transition,” “hypoxia,” and “glycolysis” was enriched in the high-risk group. It indicates that the high-risk group was bound up with the process of tumor proliferation.

Chemotherapy and ICB therapy are crucial adjunctive therapies for glioma. Temozolomide is the first-line drug for glioma treatment. As expected, the patients in the high-risk group are more sensitive to temozolomide therapy than those in the low-risk group. In addition, patients in the high-risk group have a better response to anti-PD-1 therapy. The result is consistent with our findings.

Autophagy is a conserved, self-degradation pathway that is critical for survival, differentiation, development, and homeostasis (Levine and Kroemer, 2008; Onorati et al., 2018). Although ferroptosis is distinct from other types of regulated cell death, activation of autophagy is necessary for the induction of ferroptosis under given conditions (Kang and Tang, 2017; Liu et al., 2020). In this study, according to GO and KEGG analyses, we first verified that the prognostic model has a significant correlation with most ARGs. Studies have proved that hypoxia can promote cell proliferation in tumors and the progression of tumor conversion to the malignant phenotype (Sun et al., 2021). Meanwhile, hypoxia can also protect macrophages from ferroptosis (Fuhrmann et al., 2020). We subsequently explored the relationship between the prognostic model and the hypoxia score. The results revealed that the prediction ability significantly improved with a combination of the risk score and hypoxia score. The aforementioned results contribute a new insight into the multitargeted therapy in LGG.

However, some limitations in this study should be considered. First, as a validating dataset, it had a lack of corresponding clinicopathological data in the Rembrandt database. Second, this study is based on bioinformatics analysis, and the experimental verification is needed in the future. Last, the transformation of the basic scientific advances into efficient therapeutics should be explored, which will be a formidable challenge.

## Conclusion

According to the prognostic FRGs from the three datasets, we clustered LGG patients into three subgroups which exhibited different outcomes, clinicopathological features, immune landscape, and biological processes. Subsequently, a novel clinically applicable ferroptosis-related prognostic model was constructed to benefit individualized prediction of diagnosis, treatment, prognosis, and recurrence. Moreover, our study has provided several novel insights into the connection between ferroptosis and the immunosuppressive microenvironment in LGG, which may be beneficial in individualized treatment strategies.

## Data Availability

The original contributions presented in the study are included in the article/Supplementary Material, further inquiries can be directed to the corresponding authors.
