# Supplementary material for: Characterization of the Ferroptosis-Related Genes for Prognosis and Immune Infiltration in Low-Grade Glioma
Source: Front Genet. 2022 Apr 26;13:880864. doi: 10.3389/fgene.2022.880864 (PMC9086515; doi:10.3389/fgene.2022.880864)
Supplement: Supplementary file 2 [file Table1.DOCX]

**Supplementary Material**

**Supplementary Figures**


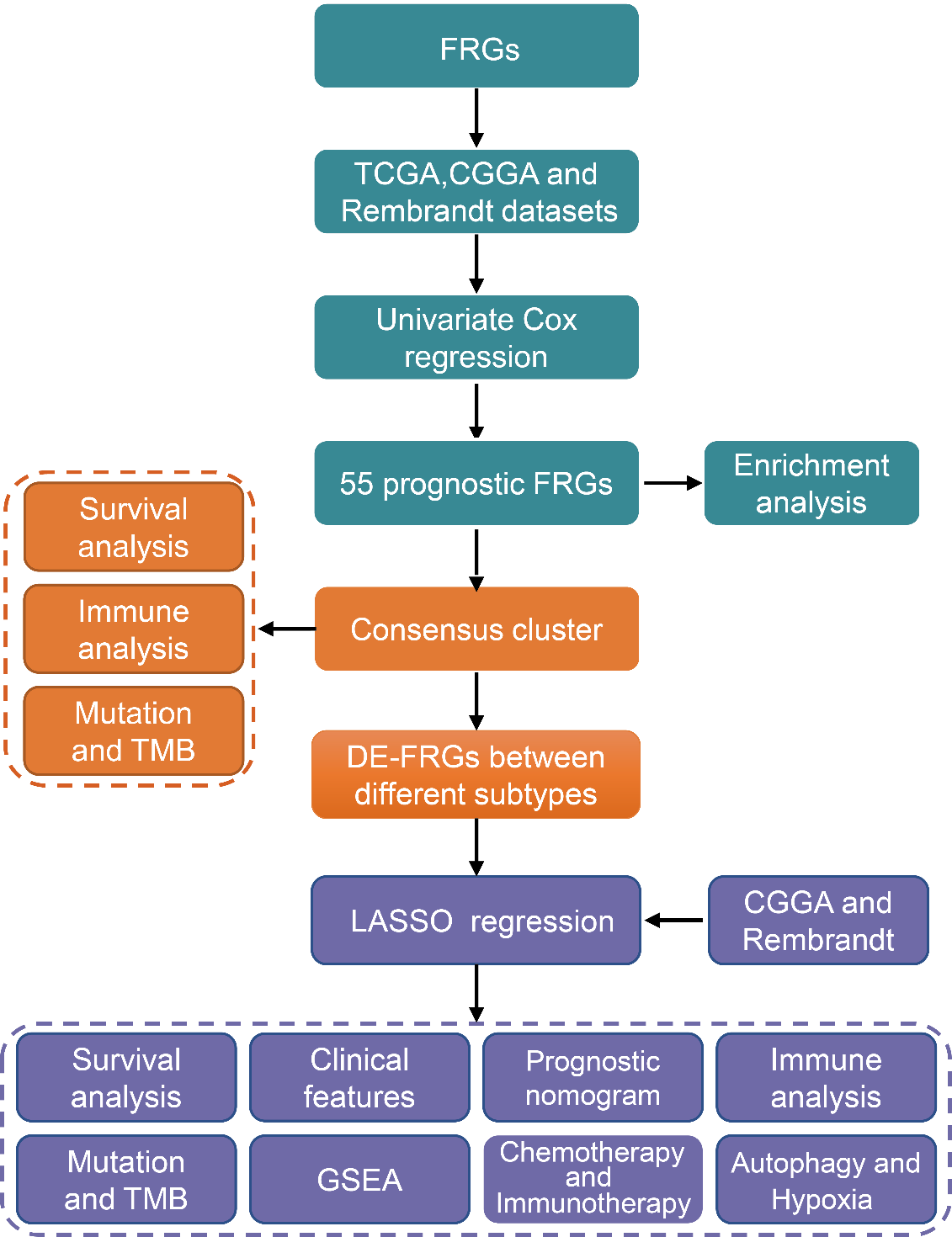


**Figure S1**. Flow chart of the study.

**
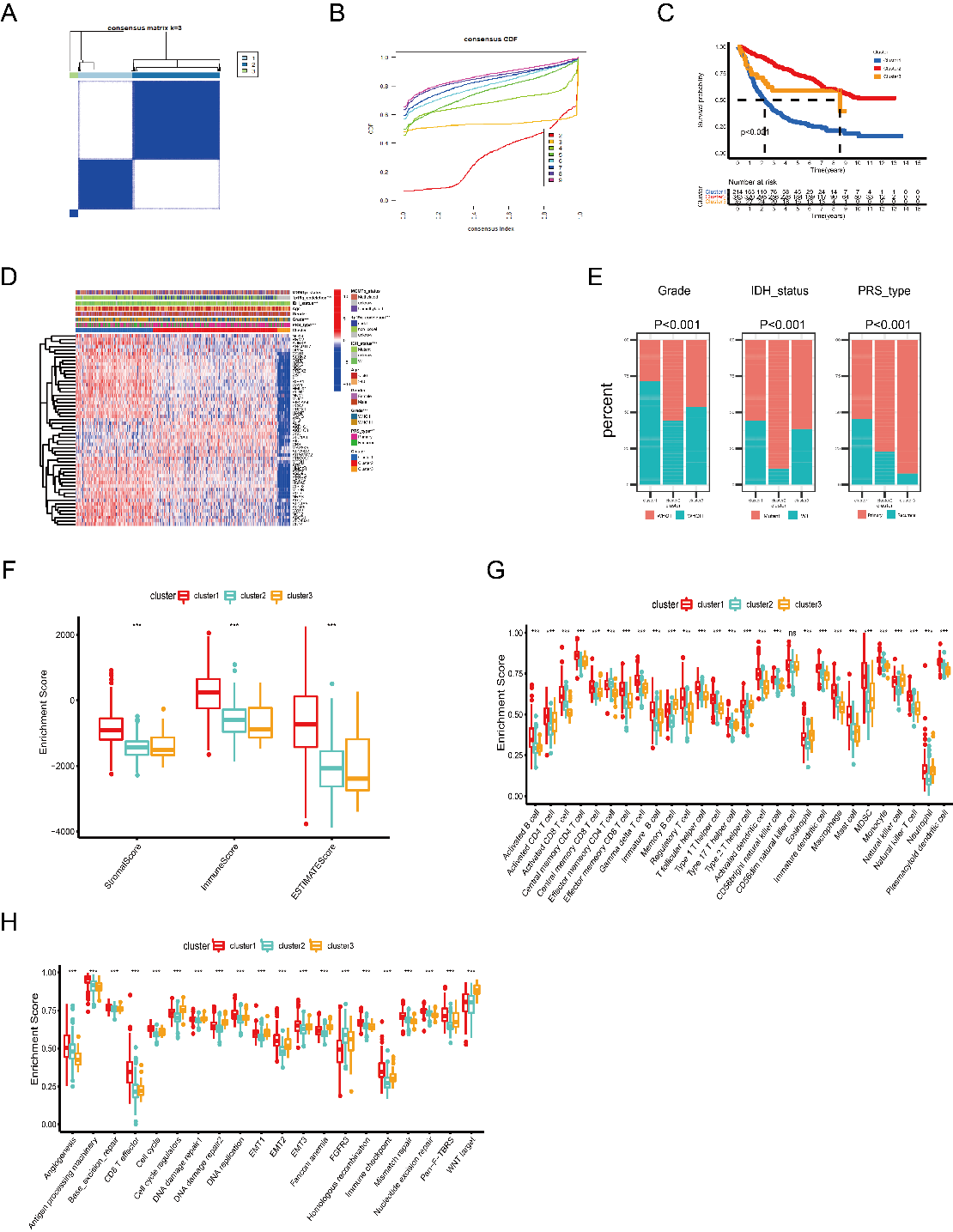
**

**Figure S2**. Differential clinicopathological features and immune landscape of LGG among different clusters in CGGA dataset.

(A) Consensus clustering matrix for k = 3. (B) Cumulative distribution function curves for k = 2 - 9. (C) Kaplan-Meier curve of overall survival among three clusters. (D) Heatmap and clinicopathologic features of the three clusters. (E) The proportion of clinical characteristics in 3 clusters. (F-H) Distribution of immuneScore (F), 28 immune cells (G), and typical biology pathways (H) across the 3 ferroptosis subtypes. The horizontal line of the box plot represented the median values (*p < 0.05, **p < 0.01, ***p < 0.001 and ns, non-significant).


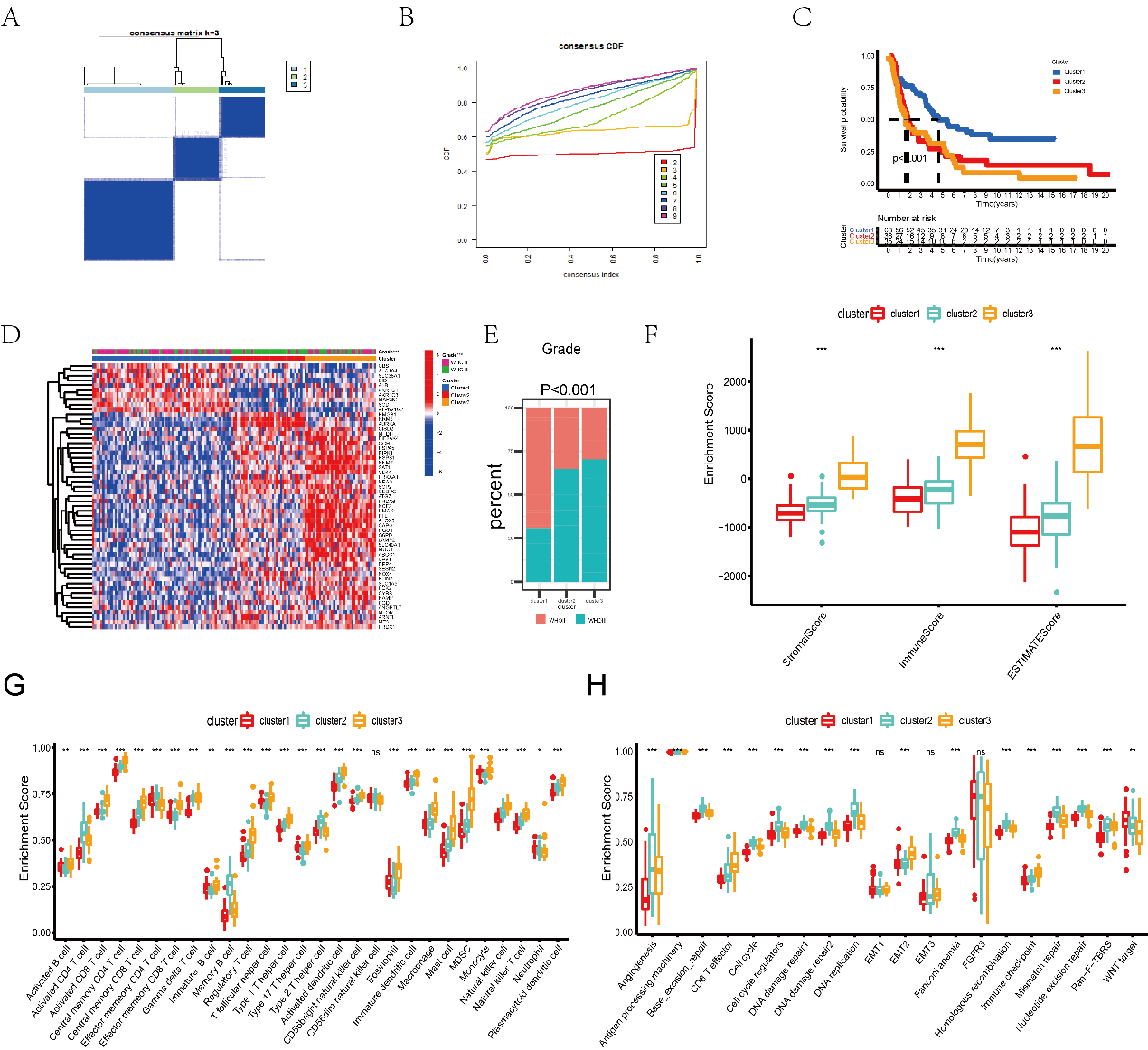


**Figure S3**. Differential clinicopathological features and immune landscape of LGG among different clusters in Rembrandt dataset.

(A) Consensus clustering matrix for k = 3. (B) Cumulative distribution function curves for k = 2 - 9. (C) Kaplan-Meier curve of overall survival among three clusters. (D) Heatmap and clinicopathologic features of the three clusters. (E) The proportion of clinical characteristics in 3 clusters. (F-H) Distribution of immuneScore (F), 28 immune cells (G), and typical biology pathways (H) across the 3 ferroptosis subtypes. The horizontal line of the box plot represented the median values (*p < 0.05, **p < 0.01, ***p < 0.001 and ns, non-significant).


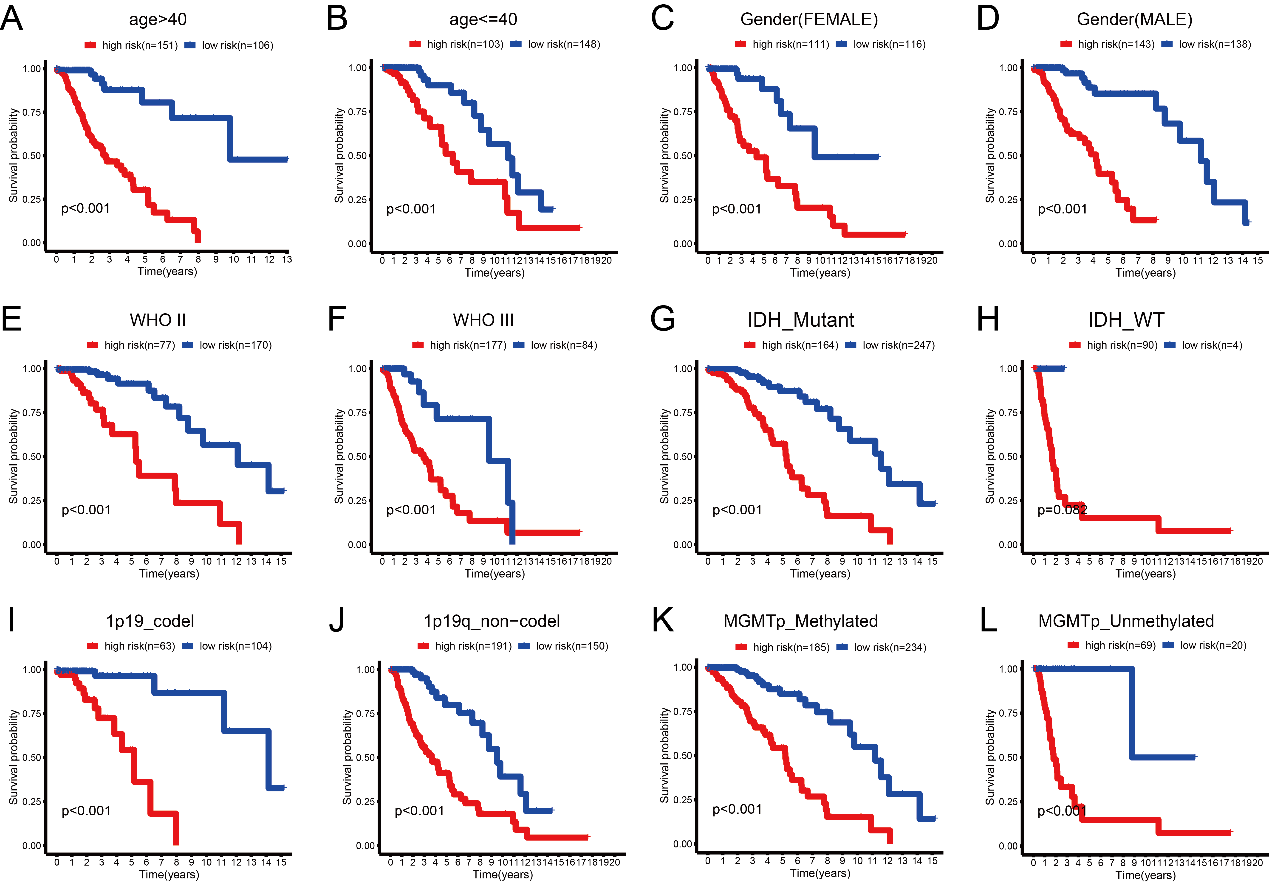


**Figure S4**. Kaplan–Meier survival curves for the low- and high-risk groups stratified by clinicopathological variables in the CGGA dataset.

(A, B) Age. (C, D) Gender. (E, F) WHO grade. (G, H) IDH_status. (I,J) 1p19q_status. (K, L) MGMTp_status.


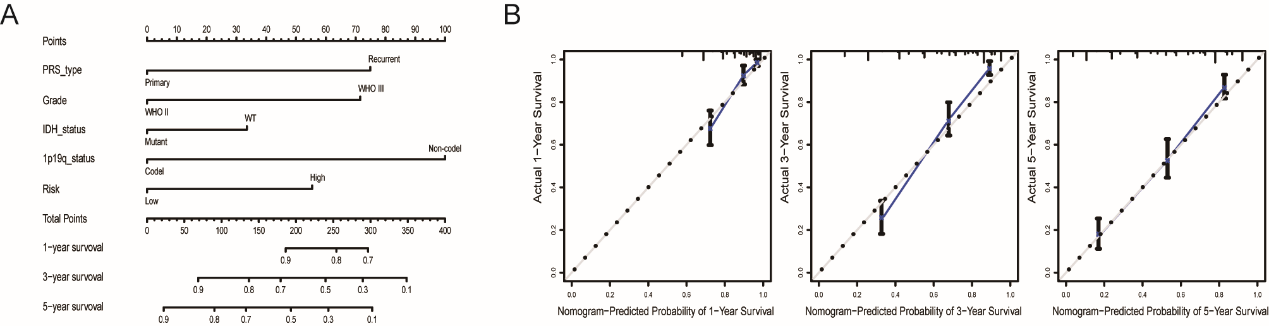


**Figure S5**. Development of a nomogram by integrating the risk score and clinicopathological characters in the CCGA cohort.

(A) Nomogram constructed to predict OS rates at 1, 3, and 5 years. (B) The calibration curves predicted 1-, 3-, and 5-year survival.


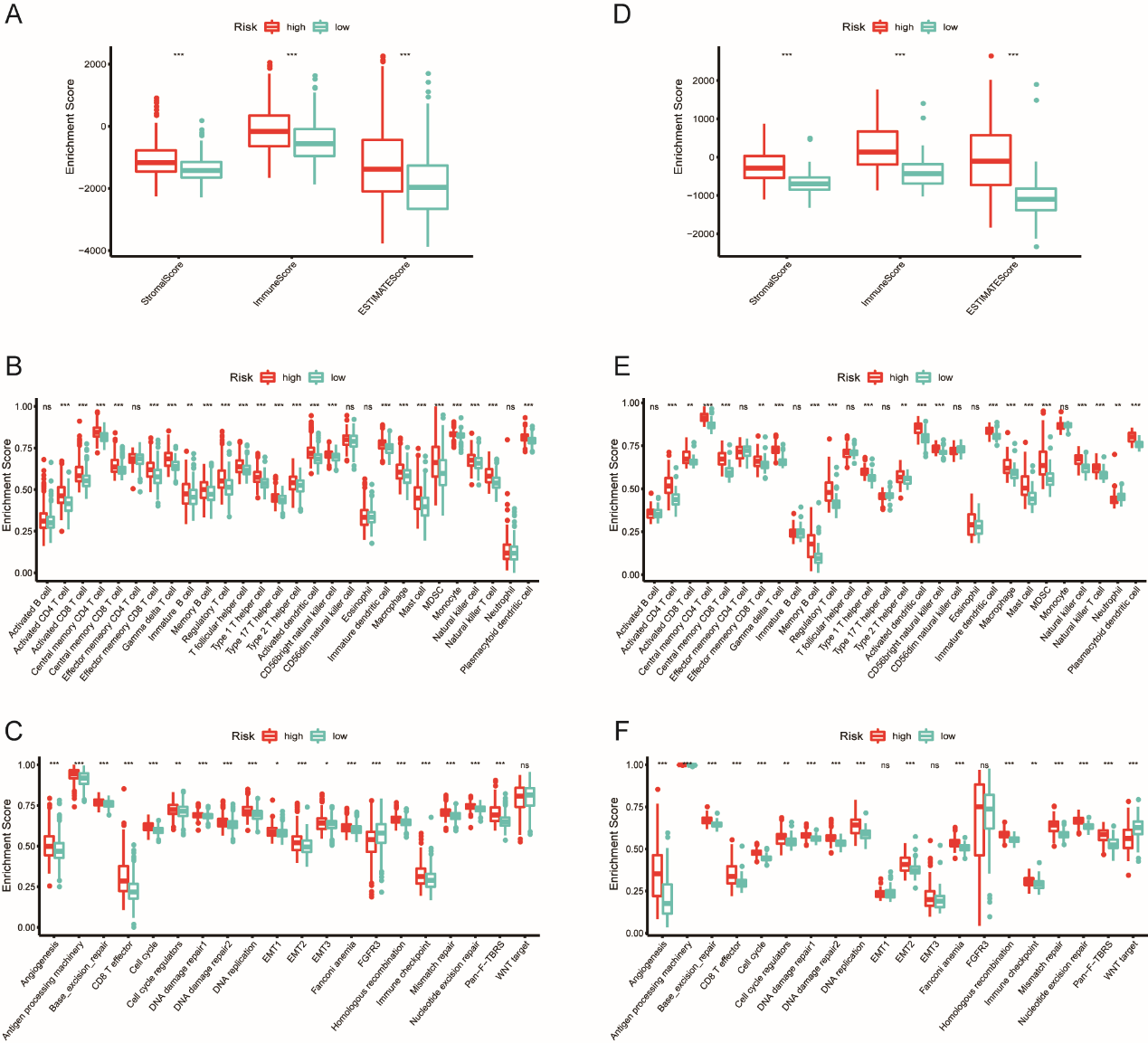
**Figure S6**. Immune microenvironment and biological process characteristics among distinct risk groups in the CGGA and Rembrandt datasets.

(A-C) Distribution of immuneScore (A), 28 immune cells (B), and typical biology pathways (C) between the high- and low-risk groups in the CGGA dataset. (D-F) Distribution of immuneScore (D), 28 immune cells (E), and typical biology pathways (F) between the high- and low-risk groups in the Rembrandt dataset. The horizontal line of the box plot represented the median values (*p < 0.05, **p < 0.01, ***p < 0.001 and ns, non-significant).
