# Supplementary material for: Characterization of the Ferroptosis-Related Genes for Prognosis and Immune Infiltration in Low-Grade Glioma
Source: Front Genet. 2022 Apr 26;13:880864. doi: 10.3389/fgene.2022.880864 (PMC9086515; doi:10.3389/fgene.2022.880864)
Supplement: Supplementary file 4 [file Table2.DOCX]

|  | Characteristics | TCGA | CGGA | Rembrandt |
| --- | --- | --- | --- | --- |

|  |  | (n=508) | (n=592) | (n=139) |
| --- | --- | --- | --- | --- |
| Age(years) | <=40 | 251 | 307 |  |
|  | >40 | 257 | 284 |  |
|  | NA | 0 | 1 | 139 |
| Gender | Female | 227 | 251 | 37 |
|  | Male | 281 | 341 | 57 |
|  | NA | 0 | 0 | 45 |
| Grade | WHO II | 247 | 270 | 70 |
|  | WHO III | 261 | 322 | 69 |
| IDH status | Mutant | 411 | 415 |  |
|  | WT | 94 | 138 |  |
|  | NA | 3 | 39 | 139 |
| 1p19q | codel | 167 | 180 |  |
| codeletion | non-codel | 341 | 372 |  |
|  | NA | 0 | 40 | 139 |
| MGMTp status | Methylated | 419 | 285 |  |
|  | Unmethylated | 89 | 200 |  |
|  | NA | 0 | 107 | 139 |

**Table S2**. Characteristics of patients in TCGA, CGGA, and Rembrandt datasets. IDH mutant status (IDH status); IDH mutant (Mutant); IDH wildtype (WT); 1p19q co-deletion (codel); 1p19q without co-deletion (non-codel); MGMTp methylated (Methylated); MGMTp unmethylated (Unmethylated).
